# UHPC Viability for Nuclear Storage Facilities: Synthesis and Critical Review of Durability, Thermal, and Nuclear Properties for Informed Mix Modifications

**DOI:** 10.3390/ma18020430

**Published:** 2025-01-17

**Authors:** Nataliia Igrashkina, Mohamed A. Moustafa

**Affiliations:** 1 Department of Civil and Environmental Engineering, University of Nevada, Reno, NV 89557, USA; nigrashkina@unr.edu; 2 Division of Engineering, New York University Abu Dhabi, Abu Dhabi P.O. Box 129188, United Arab Emirates

**Keywords:** UHPC, concrete degradation mechanisms, nuclear damage, radiation attenuation

## Abstract

Spent nuclear fuel (SNF) from the United States’ nuclear power plants has been placed in dry cask storage systems since the 1980s. Due to the lack of a clear path for permanent geological repository for SNF, consolidated and long-term storage solutions that use durable concrete and avoid current aging and licensing challenges are becoming indispensable. Ultra-high-performance concrete (UHPC) is a rapidly growing advanced concrete solution with superior mechanical and durability properties that can help realize future resilient nuclear storage facilities. Thus, the overall goal of this review study is to demonstrate the viability of UHPC as a long-term solution for future nuclear storage facilities. The paper first identifies all possible non-nuclear (environmental) and nuclear (thermal and radiation-induced) degradation mechanisms in concrete overpacks and storage modules with critical assessment and projections on UHPC performance in comparison to current conventional concrete solutions. Next, since concrete serves as a shielding material in nuclear settings, the preliminary attenuation properties of UHPC from emerging studies are synthesized along with the possible mix modifications to improve its attenuation performance. The paper identifies the major knowledge gaps to inform future research and development, aimed at rethinking the design of SNF dry storage facilities by incorporating UHPC.

## 1. Introduction

In nuclear power plants (NPPs), spent fuel assemblies, consisting of dozens or hundreds of fuel rods each, after being used and removed from the reactor core, are stored in spent fuel pools. Spent nuclear fuel (SNF), once removed, has high heat generation and is intensely radioactive. Spent fuel pools host increasing amounts of SNF with newer racks capable of holding denser arrangements of assemblies [1]. While scientists expressed their concerns regarding densely packed SNF, the U.S. Nuclear Regulatory Commission (NRC) considers wet storage as a safe and compliant means of storing fuel [2]. As spent fuel pools reached capacity in the 1980s, reactor sites began transitioning to dry cask storage systems (DCSSs) at independent spent fuel storage installations (ISFSIs) that are licensed and regulated by the Code of Federal Regulations [3]. As of February 2023, there are a total of 84 ISFSIs in the U.S., present in 36 States [4]. License applications for dry storage systems should be accompanied by a safety analysis report that includes appropriate safety evaluations. The design of the DCSS must prioritize public health and safety by ensuring adequate radiation shielding, subcriticality control, passive cooling, confinement, and structural integrity. These safety functions must be maintained under normal operating conditions, as well as off-normal and accident conditions, as detailed in NUREG-2215 [5].

There are many existing DCSSs developed by various commercial vendors designed to hold approximately 2 to 6 dozen spent fuel assemblies [6,7]. The assemblies are placed within a basket of a honeycomb structure that is either integrated into the cask itself or into a metal cylinder, referred to as a canister. Both casks and canisters, after being loaded in spent fuel pools, are dried, evacuated, filled with inert gas, and sealed. Transporting or storing canisters requires the use of appropriate shielding structures, whether oriented horizontally or vertically. These structures are known by various terms, including casks, modules, or overpacks. In the United States, the DCSSs are placed on thick reinforced concrete pads or mats [8] to reduce the chance of tip-over and provide support during seismic events. While current DCSSs are for storage or dual storage and transportation use, they can also be utilized for disposal and have been under vendors’ development [9,10].

Before a spent fuel dry storage system can be operated in the U.S., its design must be licensed by the NRC. This license initially certifies the system for a period of 20 years. Loaded dry cask storage systems need to be deemed safe and meet the technical requirements of NUREG-1927 [11] for another licensing period up to 40 years. As part of this procedure, either time-limited aging analyses or aging management programs are required, which heavily include concrete structures such as SNF dry storage facilities, and which motivate this critical review study. It is noted that aging management review requires the inspection of DCSSs, which is challenging due to safe accessibility and limited appropriate monitoring methods [12,13]. Moreover, age management of SNF dry storage is becoming more critical nowadays after the permanent repository project in Yucca Mountain was halted in the early 2010s. Given that no existing nuclear reactor can store all the SNF it will produce, especially anticipating reactor license extensions [10], the current practice of on-site storage in DCSSs can be considered a temporary solution. It is anticipated that all SNF will eventually be transferred to a permanent geological repository or consolidated storage sites. Currently, the NRC is reviewing two applications for consolidated interim storage facilities in Andrew County, Texas, and in Lea County, New Mexico [14]. While this process might require decades [1,9], the current status and aging risks of dry storage facilities, especially the concrete structures as outlined in this paper, call for rethinking the future of our nuclear structures with a focus on durability and longevity.

**Figure 1:**
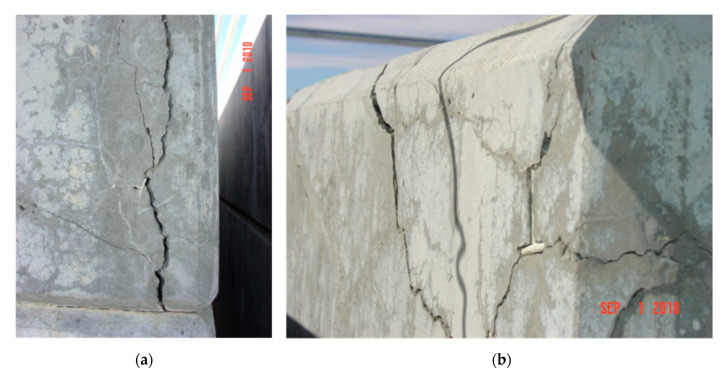
Pre-repair condition of horizontal storage module HSM 05 at Three Mile Island Unit 2 ISFSI showing damage to (**a**) northwest and (**b**) northeast roof corner (reprinted from Ref. [16]).

**Figure 2:**
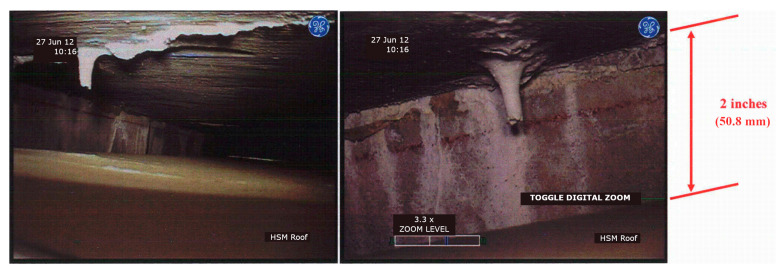
Inside view of a concrete ceiling in horizontal storage module at the Calvert Cliffs Nuclear Power Plant ISFSI (adapted from Ref. [18]).

One of the commonly used materials for shielding the outer cask for canister-based designs is concrete. The typical thickness of concrete overpacks is in the range of 0.6 to 0.9 m (2 to 3 ft) [15]. Depending on a specific design, concrete may or may not have a metallic liner and reinforcing steel. Cost considerations are driving the cask industry toward concrete designs away from all-metal cask designs [9]. The combination of prolonged service life and exposure to environmental factors can lead to degradation of concrete. Limited NRC inspections conducted since the 2000s have revealed instances of such degradation in DCSSs [15], with a prominent example being the damage caused by freeze–thaw cycles to horizontal concrete modules at Three Mile Island Unit 2 [16]. Figure 1 presents two examples of the damage observed in one of the horizontal storage modules (HSM 05) prior to repair. During the NRC inspection of ISFSI at Arkansas Nuclear One in 2003, shrinkage cracks were identified in 23 out of 24 casks of the Ventilated Storage Cask System [17]. Concrete defects were greater than ¼ in (6.35 mm) deep and ½ in (12.7 mm) wide. Signs of concrete degradation were also found during visual inspection of two horizontal storage modules located in Calvert Cliffs Nuclear Power Plant in 2012 [18]. Using remote cameras inserted through the outlet vents, formations resembling stalactites were identified on the interior surfaces of the modules (see Figure 2). These formations are likely the result of the leaching of calcium compounds, suggesting water intrusion through the outlet vent stack.

Based on the preceding discussion, a unique durable multi-purpose cask design, ideally, that can be used in a permanent disposal facility and adopted by reactor operators to avoid repackaging issues is a forward-looking solution. Extending the operational lifespan of storage casks is crucial for minimizing the need for costly and complex license renewals. Current cask designs often face maintenance challenges due to limited access and high radiation in dry storage systems. Both vendors and reactor operators seek innovative solutions prioritizing longevity and cost-effectiveness. This serves as a motivation of this paper to look into the viability of future durable concrete materials, or in particular ultra-high-performance concrete (UHPC).

UHPC is a rapidly spreading concrete material with advanced mechanical and durability properties. A couple of key research studies should be mentioned in the history of UHPC. The roots of the material can be traced back to the development of macro-defect-free cement in the 1980s by Birchall et al. [19]. The main motivation of the authors was to improve the flexural strength of cement without the need for special high-pressure treatment and fiber reinforcement. At about the same time, Bache [20] described densified cement materials incorporating ultra-fine silica powder where a superplasticizer was used to improve workability. Richard and Cheyrezy [21] produced a reactive powder concrete in the 1990s, which eliminated coarse aggregates to improve homogeneity, included steel fibers to enhance ductility, and optimized the granular mixture. Reactive powder concrete laid the foundation for the wide development of modern UHPC.

UHPC uses the packing theory to blend cement, water, fine sand, and supplementary cementitious materials such as silica fume, admixtures, and fibers (mostly steel). The high strength of the material is due to a low porosity and high packing density achieved by lowering the water/cement ratio to between 0.2 and 0.3 and carefully applying the packing theory [22]. Table 1 presents examples of compositions for both conventional concrete, or normal strength concrete (NSC), and UHPC. In contrast to NSC, UHPC usually eliminates the use of coarse aggregates to improve the homogeneity of its microstructure [23]. The goal of superplasticizers in UHPC is to improve the workability of the mix by liquefying cement and other fine particles since a higher packing density ends up in pores between the particles becoming smaller, bringing difficulties for water to lubricate the particles [24]. The summarized key properties of both NSC and UHPC are presented in Table 2. As can be seen, UHPC exhibits superior properties compared to NSC.

**Table 1:** Typical compositions of NSC and UHPC (data from [25,26]).

Example Composition	NSC	UHPC
Component: amount, kg/m^3^	Cement: 370 – Water: 204 10 mm coarse aggregate: 369 20 mm coarse aggregate: 738 Sand: 679 – –	Cement: 1050 Silica fume: 268 Water: 180 – – Sand: 514 Steel fibers: 858 Superplasticizer: 44
Water/cement ratio	0.55	0.20
Water/binder ratio	–	0.16

**Table 2:** Properties of NSC and UHPC (data from [27,28]).

Properties	NSC	UHPC
Compressive strength, MPa	<55	124–207
Flexural strength, MPa	<4.6	17–41
Shear strength, MPa	<1.2	>4.1
Direct tension, MPa	<3	Up to 10
Modulus of elasticity, GPA	20–40	40–55
Ultimate compressive strain	0.003	0.015–0.03

UHPC structural members can have reduced cross-section dimensions in comparison with members made of NSC while providing superior performance [29,30,31]. This results in a significant reduction in material usage, leading to lighter structures. UHPC has also been shown to improve the seismic performance of different structural components in buildings and bridges [32,33,34]. Nowadays, UHPC applications are gaining popularity in both research and industry and have been implemented in hundreds of projects in North America, Europe, Asia, and Australia [35]. Mainly bridge construction has utilized this material in the fabrication of structural members including girders and deck panels, as well as in field joints. Because existing codes for NSC do not fully apply to UHPC, there is a need for the development of design guidelines and recommendations. As reported by Akeed et al. [36], several nations have already been working on it, including France, Germany, Switzerland, Australia, Canada, Spain, Japan, and recently, the United States as well.

Despite its significant advantages, UHPC has some disadvantages such as the higher initial material cost or proprietary nature of robust mixtures. Nevertheless, there is significant progress in alleviating such challenges through research and advancement in developing environmentally benign UHPC at a reasonable cost [36,37]. Since UHPC, due to its dense microstructure, has a higher durability than NSC [38], this can manifest itself in saving costs in the long-term maintenance of the structure [39]. Working with UHPC often requires specialized expertise and equipment. Ensuring consistent quality control can be more challenging than for NSC, requiring careful monitoring throughout the production and placement process. There are currently no widely accepted codes or standards specifically for the design and construction of nuclear structures using UHPC. This can create uncertainty and challenges in the design and approval process.

Incorporation of new concrete materials like UHPC into cask designs can help achieve the desired future goals of dry storage facilities or even the rehabilitation and life extension of existing ones. Research and development of solutions of long-term concrete performance for nuclear applications is welcomed and supported by the industry and the Department of Energy. However, only limited work has been undertaken specifically for concrete in DCSSs as noted during a recent NRC expert panel workshop [15]. The exceptional mechanical properties, serviceability, and durability of UHPC make it a promising candidate in nuclear applications. Other future benefits of UHPC could be reduced cross-sectional dimensions and less reinforcement for the dry storage systems, making them faster and lighter for precast construction and transportation. This research is driven by the vision of a new generation of UHPC overpacks that are exceptionally durable, cost-effective, and compact, while ensuring all safety functions and minimizing maintenance requirements.

**Figure 3:**
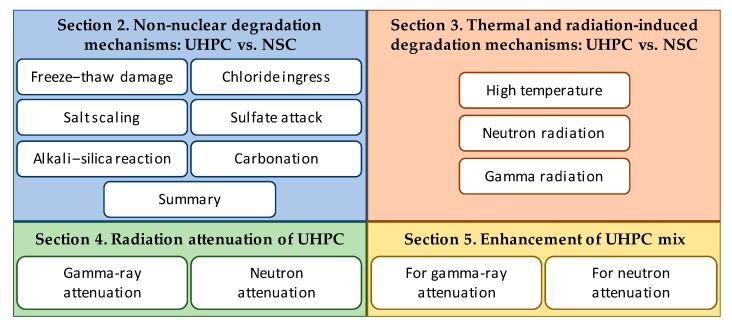
Organization of the paper and structure.

Based on the above, the evaluation of potential degradation mechanisms in typical SNF dry storage systems during the design process is crucial to ensure their long-term performance since preventing material degradation (see Figure 1 and Figure 2 for examples) can significantly extend the service life. The overall goal of this study is to demonstrate the viability of UHPC as a long-term solution for future nuclear storage facilities. As such, the specific objectives of this comprehensive review research are the following: (1) identify the potential degradation mechanisms and their underlying causes as well as their effects for concrete structures in dry cask storage systems; (2) synthesize and assess UHPC performance under the identified aging and degradation effects in comparison to conventional concrete; and (3) identify the knowledge gaps in understanding the behavior of the UHPC material under the discussed effects and potential mixture modifications for future research and development. The discussion is divided into four major sections. The first two sections (Section 2 and Section 3) focus on the general environmental non-nuclear degradation mechanisms as opposed to the more nuclear-specific thermal and radiation-induced degradation. Next, because concrete structures in cask systems are used for both structural integrity as well as radiation shielding, Section 4 and Section 5 review the emerging literature on UHPC radiation attenuation and the possible concrete additives and modifications to improve both gamma and neutron radiation shielding performance. The paper concludes with proposed theoretical UHPC mix adjustments for future SNF storage facilities; however, the results can inform larger concrete applications in NPPs. Figure 3 presents a diagram of the paper structure.

## 2. DCSS Non-Nuclear Degradation Mechanisms and Comparative Performance of UHPC and Conventional Concrete

A detailed review of the general and specific degradation mechanisms of conventional concrete used in NPPs was performed by Rasheed et al. [40]. It was summarized that concrete damage occurs by physical and chemical methods and involves loss of strength and increase in porosity and permeability. Degradation mechanisms in DCSSs can be considered a special case of those presented in buildings and structures of NPPs; for example, these mechanisms do not include damage from the operation of mechanical equipment. Mechanisms specific to DCSSs were outlined during the NRC Expert Panel Workshop [15] and in the Electric Power Research Institute EPRI Technical report [12]. Some of these mechanisms relate to the nuclear environment (e.g., radiation-induced degradation), while others are more general (e.g., alkali–silica reaction). In this section, non-nuclear degradation mechanisms of DCSSs are discussed with projections on how UHPC is likely to perform relative to conventional concrete based on synthesized durability characteristics.

### 2.1. Freeze–Thaw Damage

**Figure 4:**
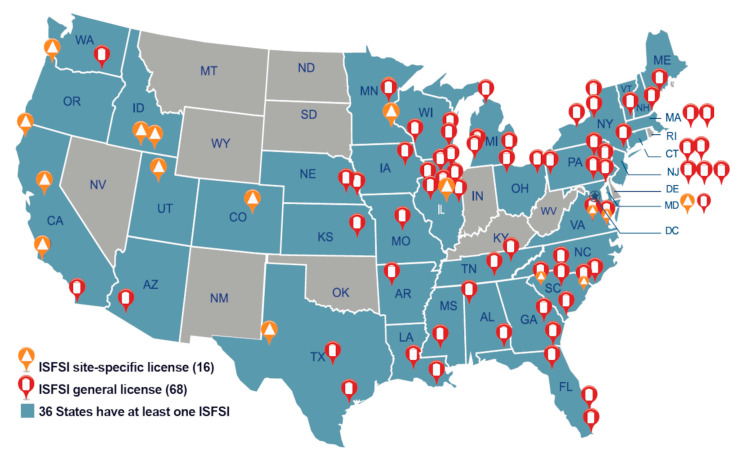
Locations of the independent spent fuel storage installations in the U.S. (reprinted from Ref. [4]).

Freeze–thaw damage is one of the possible site-specific degradation mechanisms in DCSSs occurring when water in the saturated concrete expands as it freezes. It can cause concrete spalling and cracking and promote ingress of deleterious substances. From Figure 4 showing the geographical location of current SNF storage on a U.S. map, it can be seen that many of the ISFSIs are located in cold climates where this type of degradation could be critical. Because horizontal surfaces are more prone to freeze–thaw than vertical surfaces due to possible accumulation of water, horizontal storage modules are more susceptible to freeze–thaw damage [12]. ASTM C666/C666M [41] is a standard test method for determining the freeze–thaw resistance of concrete specimens in the U.S. It has two procedures: Procedure A (more aggressive) involves rapid freezing and thawing in water, and Procedure B involves rapid freezing in air and thawing in water. The test should be continued for 300 cycles or until the relative dynamic modulus of elasticity (RDM) reaches 60% of the initial modulus or if the specimen has expanded 0.10% in length.

There is no absolute prevention from freeze–thaw degradation; however, it can be minimized. Overall, concrete can resist the expansive forces caused by the freezing of water by either having an acceptably voided microstructure or a low permeability [42]. The first approach is more common for NSC, where air-entraining agent can be introduced to improve freeze–thaw resistance by creating microscopic air pockets [43]. Meanwhile, UHPC has a dense microstructure that naturally prevents water ingress, minimizing the chance of damage due to repeating freezing and thawing cycles. Lee et al. [44] compared the freeze–thaw resistance of NSC and UHPC in accordance with ASTM C666/C666M. The values of RDM for NSC specimens at 300, 600, and 1000 cycles were 75%, 55%, and 39%, respectively; for the UHPC specimens, the corresponding values were 96%, 92%, and 90%, respectively. After 1000 freeze–thaw cycles, the compressive strength reduction for NSC reached 57% compared to 6% for UHPC. It was concluded that UHPC has a superior resistance to freeze–thaw damage in comparison to NSC.

As summarized next, important mix parameters for UHPC resistance to freezing and thawing cycles can be identified. Hasnat and Ghafoori [45] have studied UHPCs made with different combinations and types of pozzolanic materials (fly ash, silica fume, and natural pozzolan), varying steel fiber contents (0%, 1%, 2%, and 3%), and shapes (straight and hooked). All samples did improve in terms of the compressive and tensile strengths after 70 freeze–thaw cycles with mass losses varying from 0.05% to 0.55%. The authors reported that the inclusion of steel fibers had a positive influence on the freeze–thaw resistance, with preference being given to the straight fibers. The best UHPC mix contained 15% fly ash and 5% silica fume as a cement replacement. Lu et al. [46] evaluated the influence extent of different factors on the freeze–thaw resistance of UHPC including the water/binder ratio, steel fibers, superplasticizer, silica fume, and fly ash. By introducing gray correlation entropy analysis, the authors concluded that even though all the factors are important for consideration, the water/binder ratio is the most significant influence factor. Decreasing the water/binder ratio from 0.23 to 0.17 reduced the concrete compressive strength loss rate from 27.5% to 7.1% after 300 freeze–thaw cycles.

Different studies evaluated the effects of different types of treatments on the UHPC’s freeze–thaw durability. Following Procedure A of ASTM C666/C666M, Graybeal and Tanesi [42] subjected steam-treated and untreated UHPC specimens to 690 freeze–thaw cycles. It was reported the RDM of the steam-treated specimens at the end of the experiment was at least 96%. For the untreated specimens, the RDM increased significantly. Ahlborn et al. [47] investigated the freeze–thaw resistance of a commercial wide-spread proprietary UHPC mix untreated and steam-treated specimens using Procedure B of ASTM C666/C666M. After 300 cycles, all specimens had a higher relative dynamic modulus (up to 2%) than at the beginning of the testing. The authors suggested that the concrete continued to hydrate through the cycles. A small increase in mass (less than 1%) and negligible change in length were also reported. Based on the research of [48], steam-cured specimens exhibit higher compressive strength, and static and dynamic moduli of elasticity compared to water-cured specimens. It was observed that after exposure to more than 1000 cycles, the characteristics of the concrete specimens improved compared to the control specimens.

### 2.2. Chloride Ingress

High atmospheric chloride ion concentrations, such as in marine environments, can cause chloride-related corrosion of reinforcement, which leads to the degradation of concrete structures. According to NUREG-2215, it is required to consider the possibility of a DCSS’s exposure to chloride-containing environments since some of the overpack designs incorporate steel reinforcement [5]. Even though extensive research has been performed on evaluating the critical chloride content in conventional reinforced concrete, no consensus exists about its value [49]. For non-prestressed concrete, ACI 318-19 [50] has prescribed limits of chloride content from 0.15% to 1% by weight of the binder depending on concrete exposure categories and classes.

There are different methods of determining the chloride ingress, which can be divided into short-term and long-term based on the duration of the test procedure [51]. A standard test method described in ASTM C1202 [52], commonly referred to as a rapid chloride permeability test (RCPT), is an example of a short-term test. This test evaluates the resistance of a concrete sample to chloride ion penetration by monitoring the amount of electrical current that passes through the sample during a 6 h period. The AASHTO T259 test [53], referred to as a chloride or salt ponding test, is a long-term test that uses a 3% sodium chloride (NaCl) solution to pond on a specimen’s top surface for 90 days. The migration of chloride ions into the specimens is then determined. Another commonly used long-term test is a bulk diffusion test, described in ASTM C1556 [54] and NT Build 443 [55], which helps to determine the apparent chloride diffusion coefficient [56].

Due to the dense microstructure, UHPC is expected to have more resistance to chloride ingress than conventional concrete. During the RCPT tests, UHPC specimens have shown a very low or negligible chloride ion penetrability, according to ASTM C1202 [22,57,58]. Graybeal [59] has reported an extremely low content of chloride ions in UHPC samples for all the curing regimes after performing chloride ponding tests. Based on the studies of [22], the apparent chloride diffusion coefficient of UHPC, determined using the bulk diffusion test in accordance with ASTM C1556 after 95 days of exposure to a 3.5% NaCl solution, is about three orders of magnitude lower than that of NSC (1.42 × 10^−14^ m^2^/s and 1.32 × 10^−11^ m^2^/s, respectively). This means that a much longer period would be required to achieve a critical concentration of chloride at the steel–cementitious material interface and initiate corrosion. As reported by [60], the depths of penetration of chlorides for UHPC and NSC were 1 mm and 23 mm, respectively, after a six-hour quick-migration test. An apparent chloride diffusion coefficient of 0.000148 cm^2^/year (4.67 × 10^−16^ m^2^/s) was calculated for Ductal^®^ specimens that were placed inside the box girder of the Sakata-Mirai Footbridge for about five years and exposed to severe corrosive environment of the Japan Sea by [61].

Regarding the critical chloride content for UHPC, a series of tests were performed by [62] to evaluate the effects of the chloride concentration in UHPC by directly mixing NaCl into the fresh mixtures. It was reported that with up to 1.5% of NaCl inclusion proportional to the amount of cement in weight, UHPC did not undergo strength losses in compressive, bending, and dynamic Young’s modulus tests. Abbas et al. [57] observed no degradation of mechanical properties (compressive, splitting tensile, and flexural strengths) of UHPC specimens with (3%) and without fibers after 6 months of exposure to 3.5% and 10% of NaCl solutions. To note, and as reported by [63], with an increase in the slag content, the chloride ion permeability of UHPC decreases. Valcuende et al. [64] reported that neither the curing regime (20 °C, 60 °C, or 90 °C) nor the fiber content (0%, 1%, or 2%) significantly impacted the apparent diffusion coefficients of the UHPC specimens after 1 year of exposure to chloride solution.

### 2.3. Salt Scaling

Salt scaling is a degradation mechanism that takes place during the freezing of saline solution on the surfaces of concrete structures. This mechanism is not analogous to freeze–thaw damage since it mostly affects overall durability rather than mechanical integrity. Salt scaling manifests itself by the flaking of the surface material. A maximum amount of damage occurs with an as-called “pessimum” concentration equal to about 3% by weight of solute [65]. Similar to freeze–thaw damage, salt scaling can be a concern for horizontal storage modules, especially located in cold climates with marine environments.

ASTM C672/C672M [66] has been a standard test method for salt scaling experiments. In this test method, a 3% calcium chloride (CaCl_2_) solution is ponded on the concrete surface. The specimens are placed in the freezing environment for 18 h followed by a 23 °C thawing environment for 6 h with this cycle repeated daily. The results are reported in terms of the visual conditions of the surface after a certain number of freeze–thaw cycles. Sometimes, the mass of the scaled-off material is measured. To note, the standard was withdrawn in 2021. Graybeal [59] performed the evaluation of salt scaling of UHPC slabs prepared with different curing regimes according to ASTM C672/C672M. After 215 cycles, no scaling was observed on the specimens. In contrast, Bonneau et al. [67] indicated a very slight scaling of the specimens with less than 30 g/m^2^ scaling residue after 50 cycles. Vernet [68] compared the salt scaling resistance of commercial UHPC and NSC specimens. After 56 cycles, the scaled-off mass loss was less than 10 g/m^2^ and more than 1000 g/m^2^ for UHPC and NSC, respectively. Schmidt and Fehling [60] have reported a similar range of difference between these concrete types.

### 2.4. Sulfate Attack

The term “sulfate attack” (or “external sulfate attack”) refers to the deterioration of concrete caused by the penetration of sulfate ions, followed by chemical interaction with concrete hydration products [69]. This interaction forms calcium- and sulfate-based compounds [15], which cause tensile stresses inside the concrete pore structure and lead to an increase in porosity and permeability, and, in worst-case scenarios, concrete cracking, spalling, and loss of strength. Sulfate ions can be present in groundwater, seawater, and rainwater. Commonly, a 5% mass sodium sulfate (Na_2_SO_4_) solution is utilized to assess the durability of concretes and cement mortars to sulfate attack. This concentration is considered a standard exposure solution by the ASTM C1012/C1012M standard test method for mortars [70] and corresponds to 33,800 ppm SO_4_^2−^. ACI 318-19 [50] evaluates conditions as upper limit (S3 exposure class) if the dissolved SO_4_^2−^ in water is greater than 10,000 ppm. Magnesium sulfate (MgSO_4_) is another type of salt that can be used for durability tests.

According to ASTM C1012/C1012M, UHPC has shown a high resistance to 5% Na_2_SO_4_ solution [71]. Salman et al. [72] compared the mechanical properties of NSC and UHPC with hybrid fibers (steel and polypropylene) after exposure to MgSO_4_ solution of 10,000 ppm. After 180 days, the maximum percentages of reduction in the compressive strength, splitting tensile strength, modulus of rupture, and modulus of elasticity of UHPC specimens cured in sulfate water relative to tap water were 10.66%, 16.96%, 20.29%, and 19.37%; the corresponding parameters for NSC were 52.29%, 55.42%, 62.12%, and 32.80%, respectively. The research of [73] has shown the superior performance of UHPC with the inclusion of steel fibers, under external sulfate attack and both normal and elevated temperatures. Up to a 13-week exposure to Na_2_SO_4_ solutions of different concentrations, the specimens were gaining compressive and tensile strength due to continuing hydration, regardless of the exposure temperature. It was also reported that an increase in the temperature tends to facilitate damage in UHPC exposed to external sulfate attack.

Bakhbergen et al. [71] studied the effect of the mix components of UHPC, without the inclusion of steel fibers and the utilization of special curing techniques, on different mechanical and durability properties under various sodium sulfate (Na_2_SO_4_) concentrations. The researchers reported the adverse effects of an increase in the water/binder ratio and decrease in the silica fume content on the concrete performance under sulfate attack. El-Dieb [74] studied the effect of steel fibers on the durability of UHPC with self-compacting characteristics and coarse aggregates in a high-sulfate (5% Na_2_SO_4_ solution) and high-temperature condition (50 °C), resembling the Gulf environment. After 6 months of immersion in sulfate solution, the compressive strength of the concrete increased relative to the initial compressive strength after 28 days (trend similar to [73]). No significant effect on sulfate resistance was reported for the inclusion of different volume fractions of steel fibers. Based on [75], the performance of cement mortar specimens prepared with Type V cement, commonly used to improve the sulfate resistance of concrete, and Type I cement blended with either 7% silica fume or 20% fly ash under sulfate attack was similar. Peng et al. [76] evaluated the sulfate attack resistance of UHPC specimens with a high volume of supplementary cementitious materials—steel slag and ultra-fine fly ash—in terms of the mass loss and anti-erosion coefficient (percent difference in compressive strength between samples cured in water and sulfate solution). After 180 days of curing in 5% Na_2_SO_4_ solution, the specimens did not undergo any loss of strength or mass.

### 2.5. Alkali–Silica Reaction

**Figure 5:**
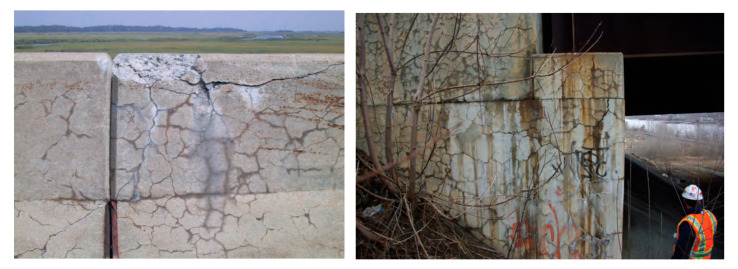
Examples of ASR cracking in bridge structures (reprinted from Ref. [79]).

Alkali–silica reaction (ASR) is referred to as the deleterious reaction that occurs in concrete between alkalis dissolved in the pore water and reactive silica in aggregates, leading to the formation of ASR hygroscopic gel [77]. Expansion of the gel creates tensile stresses, causing concrete spalling and cracking. Typically, this degradation mechanism can be identified based on characteristic map cracking on the concrete surface (see Figure 5). There are three main factors that affect the initiation of ASR: (1) presence of moisture, (2) sufficient alkali content from cementitious materials, and (3) presence of reactive silica in aggregates [78]. It takes years for ASR to manifest itself [12].

There are different adopted testing methods to assess ASR in the U.S., some of which provide severe exposure conditions to accelerate the reaction [78]. ASTM C1260 [80] is an accelerated test method that permits detection of the deleterious effects of ASR in 16 days and involves submerging mortar bars in an 80 °C sodium hydroxide (NaOH) solution. Expansion of the specimens should be recorded and evaluated; for an expansion of less than 0.10%, aggregates can be considered non-reactive [81]. ASTM C1293 [82] assesses the alkali–silica reaction by measurement of the length change in concrete prisms after one year. This test involves adding NaOH to the concrete mixing water to increase the alkali content of the mixture to 1.25% by mass of cement. An expansion of a concrete prism of less than 0.04% is considered acceptable [81].

Graybeal [59] studied the ASR of UHPC in accordance with the ASTM C1260 accelerated test method. During that project, the effect of different curing regimes was investigated. The specimens were submerged in an 80 °C NaOH solution for 4 weeks, twice the prescribed time limit, and length change measurements were recorded. After 28 days of testing, the reported expansion was 0.012%, 0.009%, 0.004%, and 0.002% for untreated, steam-treated, tempered steam-treated, and delayed steam-treated specimens, respectively, which is below the specified limit of 0.10%. It was concluded that, given its low water permeability, ASR is not a concern for UHPC under any curing regime [59]. In contrast, the studies of [83] have shown that NSC specimens have suffered expansion of 0.23%, 0.28%, and 0.36% at 14, 28, and 90 days, respectively, as well as strength degradation and visual surface damage. Möser et al. [84] evaluated the durability of pre-damaged and undamaged UHPC samples by means of a climate chamber under simulated climate conditions of Central Europe. Only a minor difference in expansion was observed between the specimens after 603 days, with all the measurements being below the threshold limit. In comparison, expansion of NSC specimens exceeded the threshold limit after 229 days of exposure.

Vernet [68] explained the UHPC resistivity to ASR due to its high silica fume content. To promote the use of more eco-friendly UHPC solutions, the following studies on ASR resistance have been performed. Based on the research of [85], 50% replacement of quartz sand by glass sand, which is a waste material, yields an environmentally friendly UHPC with satisfactory alkali–silica resistance according to ASTM C1260. It was reported that the maximum expansion of concrete specimens at 16 days was 0.03%, which is in the range of “innocuous behavior”. Abbas et al. [83] confirmed the satisfactory ASR resistance of UHPC mixtures incorporating untreated coal ash, raw slag, and locally recycled steel fibers. Through scanning electron microscopy analysis, no microcracking was detected for the tested UHPC. Finally, it was reported that the inclusion of steel fibers improved the ASR resistance of concrete.

### 2.6. Carbonation

**Figure 6:**
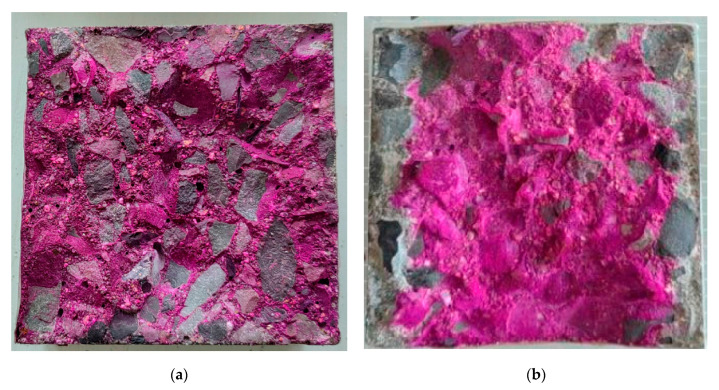
Examples of (**a**) pre-carbonized and (**b**) carbonized concrete specimens (reprinted from Ref. [89]).

Carbonation is a deleterious mechanism in concrete that can occur when atmospheric carbon dioxide (CO_2_) reacts with hydration products, such as calcium hydroxide (Ca(OH)_2_), to form calcium carbonates (CaCO_3_). Carbonation lowers the concrete pH, which may promote reinforcement corrosion and, eventually, lead to the formation of cracks, delamination, and spalling. The presence of sufficient moisture is essential for the carbonation process [40]. Also, the rate of carbonation depends on the quantity of Ca(OH)_2_ in the matrix and concentration of CO_2_ [22]. To perform accelerated carbonation tests, special chambers are utilized with a high content of CO_2_. After performing the tests, by applying a phenolphthalein solution to a concrete surface, which appears pink, if the pH level is high, the carbonation depth can be determined. If a specimen shows no color change, it can be considered carbonated (see Figure 6).

Roux et al. [86] measured the resistance of RPC200 to carbonation. Using accelerated treatment at 100% CO_2_, no carbonation was detected after 90 days of exposure. Sohail et al. [22] compared the carbonation rate of NSC and UHPC. Both types of cylinders were placed in an environmental chamber with 50% CO_2_ for six months. After six months, there were no signs of carbonation on UHPC, and a 12 mm carbonation depth was observed on NSC. The studies of Piérard et al. [87] have also confirmed the superiority of UHPC over NSC in terms of carbonation resistance: the researchers estimated the minimum concrete cover for a 100-year carbonation resistance of 65 mm for conventional concrete and 5 mm for UHPC (the specimens were exposed to a 1% CO_2_ atmosphere for one year). Valcuende et al. [64] reported no statistically significant differences in the carbonation of UHPC prepared using different curing temperatures (20 °C, 60 °C, or 90 °C) and fiber contents (0%, 1%, or 2%). Excellent carbonation resistance was observed for UHPC prepared using different waste materials as a cement substitution. Sun and Lai [88] have performed the tests using UHPC prepared with silica fume, fly ash, and slag.

### 2.7. Summary of Comparative Relevant NSC and UHPC Durability Properties

Table 3 summarizes the UHPC durability characteristics discussed in the preceding sections when compared to conventional NSC. Overall, it can be concluded that UHPC overperforms NSC in every non-nuclear durability parameter examined and identified for DCSSs, making it a viable alternative to consider for future DCSS design from a durability and longevity point of view.

**Table 3:** Comparative summary of durability characteristics of NSC and UHPC (data from [22,44,59,60,68,72,83]).

Durability Characteristic	Type of Concrete		Remarks
	NSC	UHPC	
Relative dynamic modulus, %	39	90	1000 freeze-thaw cycles, ASTM C666/C666M
Chloride ion penetration depth, mm	23	1	6 h accelerated migration test
Chloride diffusion coefficient, m^2^/s	1.32 × 10^−11^	1.42 × 10^−14^	95 days of exposure, ASTM C1556
Salt scaling mass, g/m^2^	>1000	<10	56 freeze-thaw cycles, ASTM C672/C672M
Sulfate attack: reduction, %, in - Compressive strength - Tensile strength - Modulus of rupture - Modulus of elasticity	52.29 55.42 62.12 32.80	10.66 16.96 20.29 19.37	180 days of exposure, MgSO_4_ solution of 10,000 ppm
Alkali–silica reaction expansion, %	0.28	0.012	28 days of exposure, ASTM C1260
Carbonation depth, mm	12	0	6 months, environmental chamber with 50% CO_2_

## 3. Thermal and Radiation-Induced Degradation Mechanisms and Comparative UHPC and NSC Performance in DSCC Nuclear Settings

### 3.1. Effects of Exposure to High Temperature

The concrete overpacks of DCSSs are exposed to sustained high temperature due to the decaying heat of the SNF. A DCSS utilizes a natural cooling system and heat is transferred from the cask to the environment by passive means. Thermal analysis is required by the NRC to ensure that the storage container and fuel material temperatures will remain within the allowable limits for normal, off-normal, and accident conditions. The NRS has endorsed the temperature limits of 93 °C for concrete structures in cask storages where no additional testing is required to prove the capability of elevated temperatures in normal and off-normal conditions [5]. The mechanical properties of conventional concrete can degrade at high temperatures due to dehydration of cement paste, variable thermal expansion of concrete constituents, and irreversible changes in cement and aggregates [90]. Thermal desiccation can cause drying shrinkage, which can facilitate the development of cracks. These cracks can promote the ingress of harmful environments and impair long-term structural performance. As indicated during the NRC Expert Panel Workshop, the interior surfaces of the concrete overpacks can be an area of concern because the temperatures are greater than for the outside concrete surfaces.

UHPC’s performance in elevated temperatures is somewhat different than conventional concrete. Because of its low water/binder ratio, less pore water availability, and low transport mechanisms for evaporation, drying shrinkage is minimized for UHPC [91]. According to a review summary [92], UHPC’s residual compressive strength (i.e., the specimens are cooled till room temperature after heating and then loaded till failure) vs. temperature relationship can be categorized in three distinct stages: (1) initial stabilizing and regaining stage (from room temperature to around 400 °C), (2) strength loss stage (from around 400 °C to 800 °C), and (3) total strength loss stage (after 800 °C). However, it was pointed out that the hot-temperature compressive strength (i.e., the specimens are crushed at high temperature) deteriorates gradually. The residual compressive strength of UHPC is higher than the hot-temperature compressive strength, which can be attributed to the rehydration of unhydrated cementitious materials. In the case of a hot-temperature compressive test, the coupled effect of the compressive load and the vapor pressure inside pores results in a decrease in strength. Based on the summary findings of the authors, the tensile strength and modulus of elasticity of UHPC deteriorate with the increasing temperature. As presented in [93], the increase in the steel fiber content can decrease the strength loss due to the restraining of crack propagation. Also, it was shown that the residual compressive strength reduction is slower in UHPC than in conventional concrete after heating from 200 to 500 °C.

UHPC is more vulnerable to explosive spalling than conventional concrete because of its dense internal microstructure. Moreover, an excessive quantity of silica fume decreases the spalling resistance of UHPC and changes its spalling mode [94]: the spalling products tend to pulverize into powder. It was also reported that the increasing amount of silica fume increased the spalling duration. So et al. [95] showed that the fire performance of UHPC with blast furnace slag and fly ash was superior to that of the conventional UHPC mixture with silica fume only. Several preventive measures can be used to alleviate explosive spalling [96], one of which is an inclusion of polypropylene fibers.

As discussed already, concrete structures of waste storage facilities are exposed to sustained elevated temperatures. Assuming a well-designed cooling system, it is highly unlikely that concrete will experience temperatures of greater than 400 °C, which can be considered critical for UHPC performance. For example, thermal analysis of a vertical storage cask performed by Lee et al. [97] has shown that the maximum temperature for overpack inner and outer surfaces for both off-normal and normal conditions was less than 105 °C.

### 3.2. Effects of Radiation on Concrete

The majority of the publicly available research on the effects of radiation on concrete was conducted between the 1960s and 1970s. The goal of the research was to support the development of prestressed concrete reactor vessels for high-temperature gas-cooled reactors as well as waste storage facilities [98]. Overall, concrete structures in nuclear applications are subjected to two primary forms of radiation: neutron and gamma. A state-of-the-art review of the radiation-induced degradation of conventional concrete and its components is presented in NUREG/CR-7280 [99]. The authors addressed the limitations of the collected data including the following: (1) uncertainties in experimental setups, (2) significant variability in parameters across studies, and (3) lack of applicable data to address certain parameters. There are limited neutron- and gamma-only data because tests were commonly performed with a nuclear reactor as a source where both radiations are present. A number of existing studies tested mortar specimens instead of concrete ones. As acknowledged by the report, a more complete understanding of the long-term performance of concrete in a high-radiation environment is required.

#### 3.2.1. Neutron Radiation

**Figure 7:**
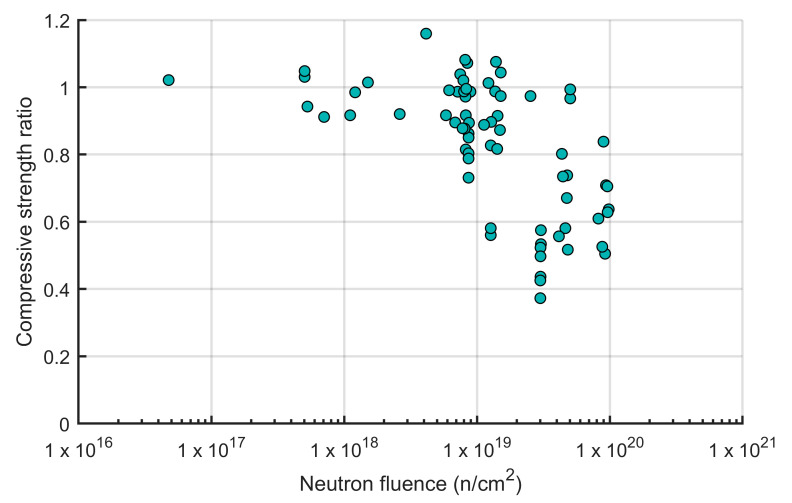
Relative compressive strength of concrete and mortar as a function of neutron fluence (adapted from Ref. [99]).

In the context of radiation-induced degradation, understanding neutron radiation is crucial due to its high penetration power. Free neutrons are released during various nuclear processes like nuclear fission in reactors and radioactive decay. These neutral particles induce ionization indirectly through interactions with atomic nuclei. The probabilities of these interactions are significantly influenced by neutron energy. While no universally accepted classification exists, a common approach, as described by [100], categorizes neutrons into three main groups based on their energy: thermal (Energy < 1 eV), epithermal (1 eV < Energy < 0.1 MeV), and fast (Energy > 0.1 MeV). The total number of neutrons that penetrate a unit area (n/cm^2^) is referred to as neutron fluence. Based on the review presented in NUREG/CR-7280 [99], at temperatures below 100 °C and fluence levels above 1 × 10^19^ n/cm^2^, concrete and mortar can experience losses in compressive strength (see Figure 7), tensile strength, and modulus of elasticity to about 40%, 20%, and 30% of the original values, respectively. All the experiments were carried out in nuclear reactors where gamma radiation was present.

It was reported that neutrons have a much larger impact on aggregates than on cement paste [99]. The authors distinguished two common groups of aggregates used in normal weight conventional concrete: siliceous (e.g., gravel and granite) and calcareous (e.g., limestone and dolomite) aggregates. From the collected data, it was observed that dimensional changes in siliceous aggregates were more pronounced than in calcareous aggregates. Field et al. [101] related swelling in siliceous aggregates to the amorphization (i.e., disordering of the crystal structure) of silicate phases. A distorted crystal structure leads to higher reactivity and increased volume of aggregates, which may make the aggregates more reactive to certain environments and cause microcracking of concrete [98]. Complete amorphization of quartz (mineral composed of silica) was observed at a fluence of 2 × 10^20^ n/cm^2^ (Energy > 0.1 MeV) [102].

#### 3.2.2. Gamma Radiation

**Figure 8:**
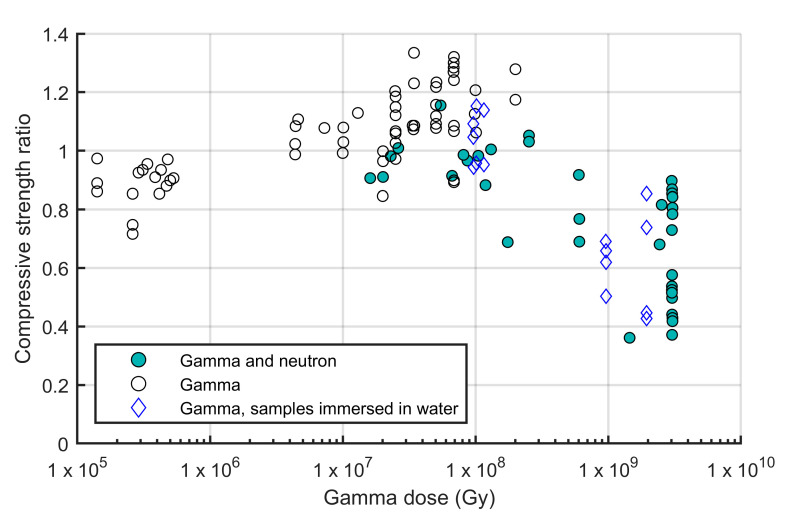
Relative compressive strength of concrete and mortar as a function of gamma dose (adapted from Ref. [99]).

Gamma rays are a form of high-energy electromagnetic radiation emitted from the nucleus of an atom during radioactive decay or other nuclear processes. The amount of gamma radiation deposited locally in matter can be specified by the absorbed dose. The SI unit of the absorbed dose is gray (Gy). Non-SI unit rad (1 rad = 0.01 Gy) can be found in the literature, predominantly in the U.S. Much of the available studies on gamma radiation of conventional concrete involve simultaneous neutron radiation [99]. Figure 8 presents the summarized by NUREG/CR-7280 results on the effects of gamma rays on the normalized compressive strength of concrete and mortar with solid symbols denoting the presence of neutron radiation. It can be seen that the compressive strength starts to decrease when the gamma dose is above 10^8^ Gy. However, in the work of Sommers [103], the only high-dose experiments where samples were subjected solely to gamma radiation (denoted as blue rhombus-shaped symbols), the concrete specimens were submerged into water. In that work, a significant reduction in the compressive strength was reported. It is not clear whether similar results can be found if the specimens were stored in dry conditions. Meanwhile, Vodák et al. [104], in a study not included in the NUREG/CR-7280 summary on gamma radiation, observed a reduction in the concrete compressive strength of about 10% under gamma radiation with a dose of 5 × 10^5^ Gy. From Figure 8, a trend of increasing relative compressive strength can be observed up to a dose of 2 × 10^8^ Gy. Maruyama et al. [105] attributed these results to gamma-induced carbonation.

Only limited data are available on the effects of gamma radiation on the tensile strength of conventional concrete without defined trends [99]. As for the elastic modulus, the trend is similar to the one discussed above for the compressive strength: the elastic modulus starts to decrease when the gamma dose is above 10^8^ Gy. Again, the majority of the experiments included neutron radiation. Possibly, because of the lack of research in the radiation-induced degradation of concrete, there is no recommended limit of gamma radiation specified in ACI 349.3R-02 [106]. One focus of the research over the years was related to radiolysis of water in the hardened cement paste under gamma radiation. As reported in [107], increasing the gamma-ray dose rates and water contents of Portland cement specimens increases the hydrogen generation rate.

#### 3.2.3. Knowledge Gaps for UHPC Radiation Behavior

The NRC staff evaluated the maximum potential accumulated neutron fluence on DCSS’s basket components after 100 years to be 2.63 × 10^16^ n/cm^2^ [108]. Reches [109] evaluated the total gamma dose expected in the walls of concrete dry casks in the order of 10^6^ Gy after 15 years in a spent fuel pool and 85 years in dry cask storage (total of 100 years). In general, concrete structures can be considered safe if cumulative radiation over their lifetime does not exceed the allowable levels for neutron fluence and gamma dose. To fully assess UHPC’s viability in waste storage facilities, it is important to understand its radiation-induced degradation mechanisms and evaluate the corresponding neutron fluences and gamma doses. Currently, to the best of the authors’ knowledge, there is no research on this topic. According to [109], gamma radiation is a dominant deleterious mechanism in dry cask storage since casks or canisters usually contain additional neutron absorbers (e.g., aluminum panels that contain boron) to prevent any possibility of criticality even if loaded with fresh fuel [110]. However, future research should evaluate the degradation mechanisms of UHPC for both neutron and gamma radiation since these results can inform larger applications in NPPs including containment buildings.

## 4. Radiation Attenuation of UHPC

Providing radiation shielding from the spent fuel in the canisters is one of the important safety functions for concrete overpacks in DCSSs [12]. To fully assess the viability of UHPC for nuclear storage, it is important to understand its attenuation properties. Attenuation is a fundamental process in radiation shielding that involves the reduction in intensity of radiation as it passes through a material. SNF emits a complex mix of radiation, including alpha particles, beta particles, gamma rays, and neutrons, each with different penetration abilities. Effective attenuation of gamma rays and neutrons ensures sufficient shielding against other radiation types [111]. For this reason, the focus is mainly placed on attenuating gamma rays and neutrons.

### 4.1. Gamma-Ray Attenuation

**Figure 9:**
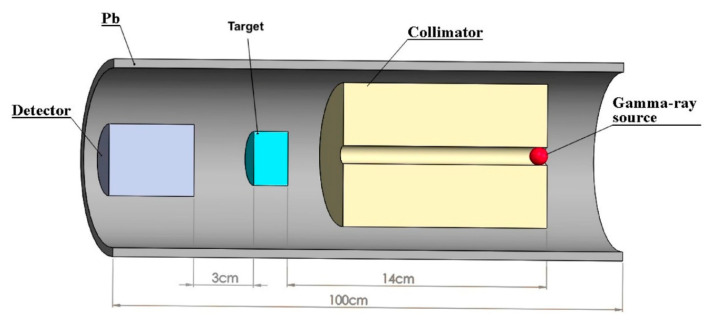
Schematic diagram of a typical gamma-ray attenuation measurement setup (reprinted from Ref. [112]).

The linear attenuation coefficient (μ) quantifies the effectiveness of a material in attenuating the photon intensity. It represents the probability of a photon interacting with the material per unit length and is typically measured in cm^−1^. Experimental determination of the coefficient involves using a narrow beam of mono-energetic photons, commonly generated by sources like cesium-137 (^137^Cs) and cobalt-60 (^60^Co). These experiments are typically conducted in specialized facilities. A schematic representation of the experimental setup is presented in Figure 9. Equation (1) relates the incident intensity ($I_{0}$) of photons emitted from a gamma-ray source to the transmitted intensity (I) measured by a detection system through the sample thickness (x).

(1) $$I=I_{0}e^{-\mu x}$$

Along with the linear attenuation coefficient, the half-value layer (HVL) and tenth-value layer (TVL) are commonly reported. These are thicknesses of any given material required to attenuate 50% and 10% of the incident intensity, respectively. The HVL and TVL are proportional to the attenuation coefficient and can be found using Equations (2) and (3), respectively. The attenuation coefficient can also be expressed as the mass attenuation coefficient $\left(\mu_{m}\right)$ as a ratio with respect to the density of the material (ρ), as shown in Equation (4).

(2) $$HVL=\frac{\ln2}{\mu }$$

(3) $$TVL=\frac{\ln10}{\mu }$$

(4) $$\mu_{m}=\frac{\mu }{\rho }$$

Software programs like WinXcom [113] and its predecessor XCOM [114] can be used to theoretically determine gamma-ray attenuation coefficients. As summarized by the authors’ previous conference paper [115], several studies have determined the linear attenuation coefficients of UHPC presented in Table 4.

**Table 4:** Summary of gamma-ray linear attenuation coefficients of UHPC reported in the literature (adapted from Ref. [115]).

Study	Linear Attenuation Coefficient (cm^−1^)		
Experiments	Gamma-ray source		
	^137^Cs		^60^Co
Azreen et al. [116]	0.155		0.096
Tufekci and Gokce [117]: Reported for different water/binder ratios of 0.18, 0.24, and 0.36	0.173		0.116
	0.177		0.117
	0.169		0.111
Rashid et al. [118]	0.146		0.091
Khan et al. [119]	0.187		−
Han et al. [120]	0.154		−
Theoretical calculations	Decay energy (MeV)		
	0.662	1.173	1.332
Gökçe et al. [121]	≈0.202	≈0.152	≈0.144

### 4.2. Neutron Attenuation

Unlike charged particles, neutrons pass through the electron cloud of an atom and interact directly with the nucleus. There are many ways in which these interactions can occur [122]. The extent of each type of interaction is described in terms of quantities known as cross-sections. The product of the atom density and the cross-section is called the macroscopic cross-section (Σ). Experimental setups for measuring neutron attenuation resemble those used for gamma-ray attenuation (see Figure 9), but with key differences in the radiation source and the need for neutron moderation. Commonly used neutron sources include plutonium–beryllium (Pu-Be) or americium–beryllium (Am-Be), which emit fast neutrons [123]. A polypropylene block is effective to moderate the fast neutrons emitted from the source, reducing their energy and producing a beam of slower thermal neutrons [124].

The removal macroscopic cross-section ($\Sigma_{R}$), typically expressed in cm^−1^, represents the probability of a fast neutron undergoing its first collision within a material. This interaction removes the neutron from the fast energy group through scattering [125]. Equation (5) relates the incident and transmitted intensities of fast neutrons ($I_{0}$ and I, respectively) to the material thickness (x).

(5) $$I=I_{0}e^{-\Sigma_{R}x}$$

Equation (5) provides only a simplified approach to the calculation of neutron attenuation, due to the complex nature of neutron interactions with nuclei. A similar equation can be applied to thermal neutrons. In addition, the half-value and tenth-value layers can be found using Equations (6) and (7), respectively. Moreover, for theoretical calculations of fast removal cross-sections, NXcom is commonly utilized [111].

(6) $$HVL=\frac{\ln2}{\Sigma_{R}}$$

(7) $$TVL=\frac{\ln10}{\Sigma_{R}}$$

While some research studies exist on the gamma-ray attenuation properties of UHPC, its neutron attenuation performance has received limited attention until recently. Arfa et al. [126] evaluated the values of macroscopic cross-sections of slow and fast neutrons in UHPC samples with different percentages of steel fiber contents and compared the results with NSC containing dolomite. For all neutron energies, the values of macroscopic cross-sections of NSC were greater than those in UHPCs. As was mentioned by the authors, this difference can be explained by the fact that the amount of free water is more in ordinary concrete, making it a better moderator for fast neutrons. For slow neutrons, the difference was explained by the presence of dolomite, and the high scattering cross-section of CaO. Steel fibers were found to be a modest factor in the neutron attenuation performance of different UHPCs. To fully realize the potential of UHPC in nuclear applications, it is essential to address the current knowledge gap regarding its neutron attenuation properties. Further research is urgently needed to characterize the neutron attenuation performance of various UHPC mix designs, including those with potential modifications.

## 5. Possible Mix Modifications of UHPC

Research on optimizing the mix design of UHPC to enhance its attenuation properties remains limited. In contrast, extensive research has been conducted on NSC for nuclear applications. Many of the findings could potentially be extended to UHPC. This section provides an overview of the studies that have considered mixture additives to improve the radiation shielding performance of UHPC, with a discussion on promising ones that can potentially enhance both gamma-ray and neutron attenuation.

### 5.1. Enhancement in Gamma-Ray Attenuation

**Figure 10:**
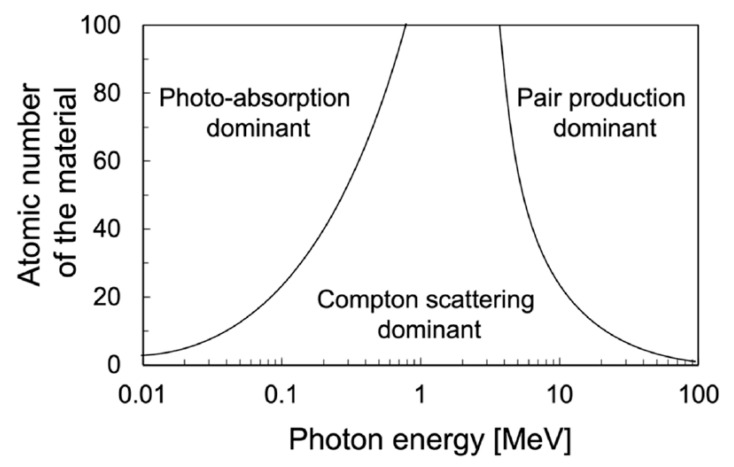
Dominance regions for the gamma-ray interaction processes (reprinted from Ref. [132]).

Gamma rays interact with matter through various processes, primarily the photoelectric effect, Compton effect (or Compton scattering), and pair production. The photoelectric effect and pair production are absorption processes where the gamma-ray photon is completely absorbed, while the Compton effect contributes to both the absorption and scattering of photons. Each of these processes involves the ejection of electrons from atoms, the details of which are described elsewhere [127]. The relative importance of three interaction processes depends on the photon energy (hv) and the atomic number (Z) of the absorber (see Figure 10). To enhance gamma-ray attenuation, increasing the electron density of the shielding material is crucial. While achieving high density through improved compaction can be challenging in NSC, due to the presence of coarse aggregates, utilizing high-density materials with elements of high atomic numbers offers a more effective approach. Extensive research has focused on developing heavyweight concrete with a density exceeding 2600 kg/m^3^ by incorporating coarse aggregates like magnetite, barite, and hematite [123,128,129,130,131]. Inspired by these studies, researchers have explored the use of heavy fine aggregates in UHPC to enhance gamma-ray attenuation.

Rashid et al. [118] reported significant improvements in the linear attenuation coefficients for UHPC containing magnetite fine aggregate when exposed to ^137^Cs and ^60^Co sources. Similarly, Han et al. [120] observed a correlation between the increasing magnetite content in UHPC and enhanced gamma-ray attenuation, with linear attenuation coefficients ranging from 0.167 to 0.202 cm^−1^. While these studies demonstrate the effectiveness of magnetite in improving the attenuation performance, they also highlight a potential trade-off with mechanical properties. Both studies reported reductions in the compressive strength for UHPC mixes containing magnetite. Rashid et al. [118] presented a 13% reduction, while Han et al. [120] found a more moderate decrease of 4.5%, when fully substituting sand with magnetite. To note, in the studies of [118], mixes with and without magnetite had similar tensile strengths (83 and 81 MPa).

Previous research has demonstrated the effectiveness of barite fine aggregate in enhancing the gamma-ray attenuation of UHPC [116,117,121]. However, the inclusion of barite can lead to reductions in the mechanical properties, such as compressive strength (15 to 21%) and, as demonstrated by Gökçe et al. [121], flexural strength (36%) and modulus of elasticity (16%). Tufekci and Gokce [117] reported that the curing regime (water or steam curing) and steel fiber content (1%, 2%, or 3%) do not affect the linear attenuation coefficients of the specimens. The results of a UHPC mix containing barite fine aggregate were higher than those of NSC incorporating barite coarse aggregate, confirming the importance of a dense microstructure for gamma-ray attenuation.

**Figure 11:**
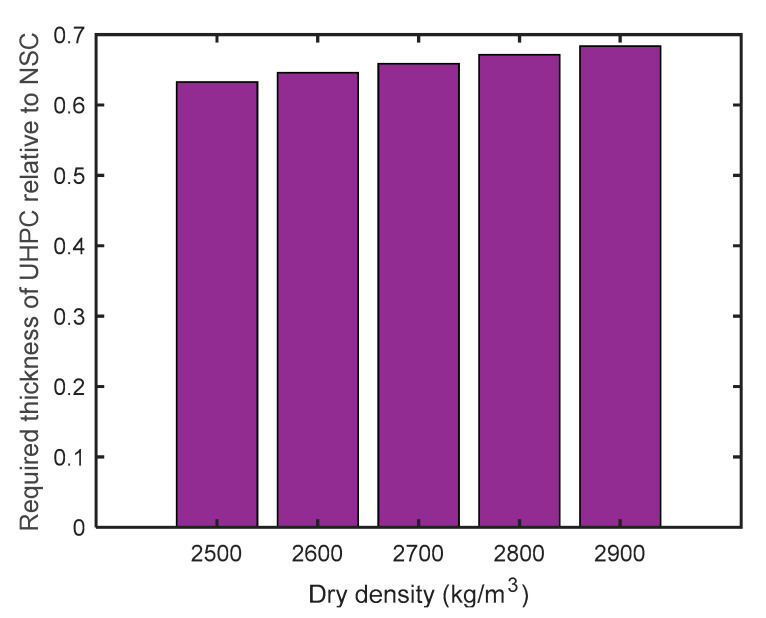
Thickness of UHPC relative to NSC required for 99% gamma-ray attenuation (adopted based on previous work from [119,131]).

For UHPC specimens containing hematite fine aggregate, Azreen et al. [116] reported linear attenuation coefficients of 0.165 cm^−1^ and 0.108 cm^−1^ for ^137^Cs and ^60^Co sources, respectively, which were higher compared to the coefficients of UHPC with sand as a fine aggregate (see Table 4). However, this enhancement in radiation attenuation was accompanied by a 10% reduction in compressive strength. Khan et al. [119] further investigated the effects of incorporating hematite powder in UHPC, considering various dosages as a partial replacement of sand. They found that the addition of hematite powder improved both the compressive and flexural strengths, increased the density, and did not adversely affect the modulus of elasticity. Furthermore, they obtained an empirical equation based on regression analysis to calculate the required thickness of UHPC for 99% attenuation of gamma rays, considering the dry density of the material. A similar equation was proposed by [131] for NSC. To compare the radiation attenuation performance of UHPC and heavyweight conventional concrete, the same range of dry densities can be used. As depicted in Figure 11, UHPC achieved a 99% attenuation of gamma rays with less thickness compared to NSC.

Evaluation of the attenuation performance of UHPC incorporating waste materials, amang and lead glass, was studied by [133]. ^137^Cs and ^60^Co sources were used for gamma attenuation tests. UHPC containing amang had the highest density and linear attenuation coefficients for both the sources. However, the authors raised a concern regarding the usage of amang in concrete because of its possible radiological effects on workers and the environment. Specimens containing lead glass experienced a reduction in compressive strength to 21% at day 610 compared to day 28. In the study [117], attenuation tests with ^137^Cs and ^60^Co sources were performed with UHPC mixes incorporating granulated ferrous waste, a by-product generated during the production of iron and steel. Using granulated ferrous waste improved the compressive strength and gamma-ray attenuation performance in comparison with UHPC containing quartz sand and quartz powder. For steam-cured specimens produced with a water/binder ratio of 0.18 and steel fiber content of 3%, the compressive strength reached 201 MPa.

To conclude, hematite fine aggregate enhances the gamma-ray attenuation of UHPC while minimizing adverse effects on its mechanical properties compared to barite and magnetite, making it a promising additive for nuclear waste storage applications. Granulated ferrous waste is another favorable additive; however, additional studies should be performed to evaluate its effects on mechanical properties other than compressive strength.

### 5.2. Enhancement in Neutron Attenuation

Fast neutrons, with their high kinetic energy, present a unique challenge in radiation shielding applications. They require a two-step attenuation process: moderation followed by capture. Moderation involves slowing down fast neutrons through a combination of inelastic scattering with heavy elements, such as iron, and elastic scattering with light elements like hydrogen [134]. Inelastic scattering excites the target nucleus, reducing the neutron’s energy, while elastic scattering transfers kinetic energy through direct collisions. Once moderated to lower energies, neutrons can be effectively captured by materials with high neutron capture cross-sections. This capture often results in the emission of capture gamma rays. Due to its large neutron capture cross-section and the emission of only low-energy capture gamma rays, boron has become a common additive in neutron shielding materials [135].

Natural aggregates like serpentine and limonite have been shown to effectively moderate neutrons in concrete applications because they both contain hydrogen [136,137]. Different studies have evaluated the neutron absorption performance of NSC containing boron [124,135,138]. Boron can be added in concrete in three different ways: as boron-containing aggregates, as boron-containing cements, and in the form of soluble compounds. Future experiments should focus on quantifying the neutron attenuation properties of UHPC with these materials. It is crucial to evaluate the effects of these modifications on other key properties, like compressive strength, flexural strength, and durability.

## 6. Concluding Remarks

In this study, the full range of general (non-nuclear) and specific (nuclear) concrete aging and degradation mechanisms in DCSSs in nuclear settings is identified and presented to provide a framework for assessing the desired material characteristics and project it for UHPC. As such, a comparative assessment of the UHPC behavior versus conventional NSC and the UHPC projected performance under the various identified degradation effects in DCSS nuclear settings are comprehensively provided in this review paper. The paper focused, for the first time, on synthesizing together the durability, thermal, and nuclear radiation and attenuation aspects to establish the viability of UHPC as a proposed solution for future consideration in SNF storage facilities. The paper concludes with extrapolating emerging knowledge on nuclear concretes to inform and propose potential mix modifications for nuclear UHPC using various concrete additives and modifications to improve the shielding performance.

The review also provides and confirms several key conclusions that are summarized as follows:

- UHPC has better long-term performance parameters and durability characteristics in response to the identified degradation mechanisms in nuclear settings than conventional concrete.
- Specific desired attributes such as drying shrinkage that results from thermal desiccation is minimized for UHPC.
- For gamma radiation, hematite and granulated ferrous waste are promising additives to be considered for the future mix design of UHPC.
- Two major knowledge gaps are identified herein and urgently recommended for future research to consider as follows: (1) understanding the radiation-induced degradation mechanisms of UHPC and allowable limits of neutron fluences and gamma doses, which can affect its mechanical properties; and (2) evaluating the neutron attenuation performance of UHPC along with the effects of the possible mix modifications on improving this aspect of behavior.

## List of Abbreviations

ACI	American Concrete Institute
ASTM	American Society for Testing and Materials
ASR	Alkali–silica reaction
DCSS	Dry cask storage system
ISFSI	Independent spent fuel storage installation
NPP	Nuclear power plant
NRC	Nuclear Regulatory Commission
NSC	Normal strength concrete
NUREG	Nuclear Regulatory Report Series
SNF	Spent nuclear fuel
RCPT	Rapid chloride permeability test
UHPS	Ultra-high-performance concrete
